# Enhanced Salt Removal of Fresh Water by Recovery-Reduced Ion Concentration Polarization Desalination

**DOI:** 10.3390/membranes14030056

**Published:** 2024-02-21

**Authors:** Myeonghyeon Cho, Seokhee Han, Seohyun Lee, Joong Bae Kim, Bumjoo Kim

**Affiliations:** 1 Department of Future Convergence Engineering, Kongju National University, Cheonan 31080, Republic of Korea; ekxm7@naver.com (M.C.); han991001@naver.com (S.H.); 2 Department of Mechanical and Automotive Engineering, Kongju National University, Cheonan 31080, Republic of Korea; seohyunlee2002@gmail.com

**Keywords:** desalination, ion concentration polarization, ion exchange membrane, electrodialysis

## Abstract

Here, we examine electromembrane systems for low-concentration desalination applicable to ultrapure water production. In addition to electrodialysis and ion concentration polarization (ICP) desalination, we propose a recovery-reduced ICP strategy for reducing the width of the desalted outlet for a higher salt removal ratio (SRR). The correlation between conductivity changes and thickness of the ion depletion zone is identified for electrodialysis, ICP_H_ (1:1), and ICP_Q_ (3:1) with a low-concentration feed solution (10 mM, 1 mM, 0.1 mM NaCl). Based on the experimental results, the scaling law and SRR for the electroconvection zone are summarized, and current efficiency (CE) and energy per ion removal (EPIR) depending on SRR are also discussed. As a result, the SRR of electrodialysis is mostly around 50%, but that of recovery-reduced ICP desalination is observed up to 99% under similar operating conditions. Moreover, at the same SRR, the CE of recovery-reduced ICP is similar to that of electrodialysis, but the EPIR is calculated to be lower than that of electrodialysis. Considering that forming an ion depletion zone up to half the channel width in the electromembrane system typically requires much power consumption, an ICP strategy that can adjust the width of the desalted outlet for high SRR can be preferable.

## 1. Introduction

Desalination is a process that removes salt ions dissolved in an aqueous electrolyte solution, which ranges from fresh water (<0.5 ppt) to brine (>50 ppt) in terms of feed salinity [1,2]. In particular, fresh water desalination needs to remove most trace salts from low-concentration feed to be applied for the production of ultrapure water. To produce pure water, reverse osmosis has been widely used to desalinate pretreated brackish water [3,4,5]. However, reverse osmosis has a limit to solely encompass the following processes to acquire ultrapure water since it cannot completely remove residual ions and organic matter in such a desalination of pure water [6,7]. Alternatively, electromembrane desalination technologies have been utilized to deal with fresh water to produce ultrapure water [8,9,10].

Electrodialysis, the most representative electromembrane desalination, has the advantages of scalable equipment and complete desalination [11,12,13]. However, in areas where the ion concentration is very low, rapid increases in electrical resistance due to concentration polarization cause increased operating costs [14,15,16]. To complement this limitation, a CEDI (Continuous Electrodeionization), a system that combines an ion exchanger (IX) and electrodialysis, has recently been used in the production of ultrapure water [6,7,8,9,17]. It would be efficient when considering only enhanced ion transfer in a low-concentration solution, but a meticulous pretreatment for feed water is essential, and the ion exchange resin filled in the fluidic channel increases flow resistance [17,18,19,20]. Therefore, to resolve the above limitations, it is necessary to keep improving and developing electromembrane desalination dealing with low-concentration feed.

**Figure 1:**
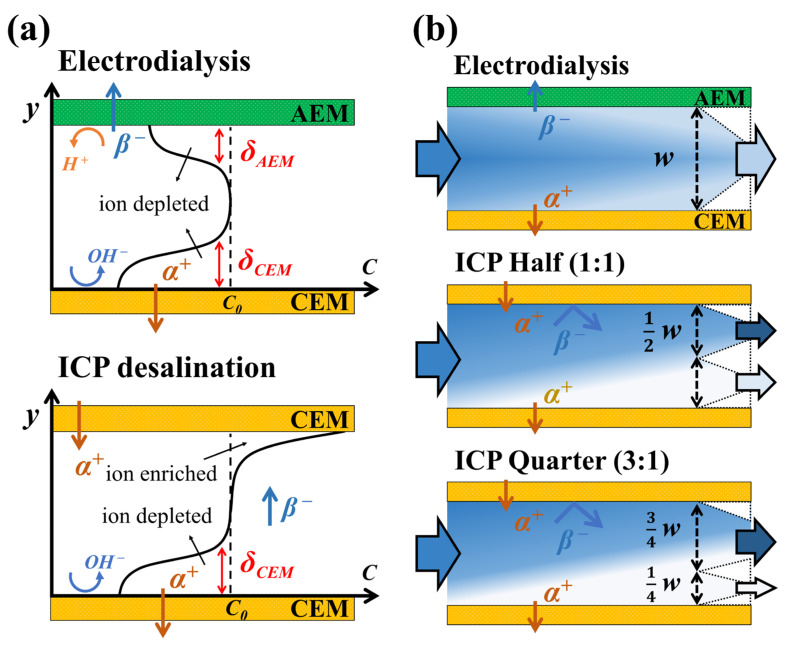
(**a**) Schematics of ion transfer and local ion concentration in electrodialysis and ICP desalination. *δ_AEM_* and *δ_CEM_* indicate the ion depletion zone on the AEM and CEM, respectively. (**b**) Schematics of electrodialysis and the recovery-reduced ICP strategy. ICP Half (ICP_H_) and ICP Quarter (ICP_Q_) have a recovery of 0.5 and 0.25, respectively, depending on the width of the desalted outlet channel.

Previously, a novel strategy of electromembrane desalination that utilizes the ion concentration polarization (ICP) phenomenon was proposed by the Han group [21,22]. Different from electrodialysis, ICP desalination, which utilizes only cation exchange membranes (CEMs) or anion exchange membranes (AEMs), separates and extracts each enriched and desalted flow as it is within one channel. (See Figure 1a). R. Kwak et al. [21] successfully demonstrated that ICP desalination has a current efficiency of more than 20% (e.g., NaCl solution on CEMs) higher than that of electrodialysis based on both experiments and numerical analysis. B. Kim et al. [22,23] demonstrated the economic feasibility of ICP desalination compared with other methods (e.g., MVR, MSF) in the separation process of high-concentration brine and also presented a simultaneous reduction in salt concentration and various charged particles (e.g., bacterial cell, crude oil emulsion) by bifurcated output flow channel via fluorescent visualization. J. Yoon et al. [24] proposed a return flow ICP desalination to enhance the desalination performance by alleviating the concentration polarization phenomenon. Accordingly, by utilizing the ICP strategy, one can expect higher current efficiency (i.e., salt removal) and fewer technical limits (e.g., pretreatment, purchasing cost, pumping loss) from additive ion exchange resin in desalting low-concentration feed. However, the application of ICP desalination to the low-concentration feed treatment is still unexplored since the preceding studies on ICP desalination have mainly focused on the medium- to high-saline (i.e., brackish to brine) feed water.

Herein, we experimentally evaluate ICP desalination by using a low-concentration feed equal to or lower than fresh water. Based on fluorescent visualization, we observe the electrohydrodynamic behavior of the ion depletion zone under various operating parameters (e.g., feed concentration, applied voltage, recovery) and compare the desalination performances of electrodialysis and ICP desalination. Basically, the relationship between the thickness of the ion depletion zone and the downstream flow should be significant since ICP desalination extracts a steady and depleted stream flow on the membrane surface, while electrodialysis recommends flow mixing to reduce the concentration gradient. Hence, we also devise a recovery-reduced ICP strategy to extract wholly depleted stream flow for a complete desalination with a low-concentration feed.

## 2. Materials and Methods

### 2.1. Background

In electrodialysis, the AEM and CEM are alternately arranged, resulting in a single desalted channel as negative and positive ions exit through the AEM and CEM, respectively [11,12,25] (See Figure 1a). In ICP desalination, only CEMs (or AEMs) are arranged, resulting in respective ion depletion and enrichment zones on the bottom and the top, which produce desalted and enriched output flow via bifurcated downstream channel [21,22,23]. ICP desalination shows better desalting performance (i.e., more salt removal) than electrodialysis due to thicker ion depletion zone on the CEM when NaCl feed used [21,22]. Furthermore, beyond limiting current density (i.e., the overlimiting regime), ICP desalination utilizing only CEMs also exhibits better desalination since increased catalytic activity of fixed charged groups in AEM degrades active electroconvection and ion transfer [26,27,28]. Given that we aim to remove salt ions as much as possible in low-concentration desalination, it is not suitable to keep the ICP strategy with evenly bifurcated channels only. Instead, we added another type that reduces the recovery of the desalted water by half (i.e., recovery of 0.25) to extract much depleted output flow. As shown in Figure 1b, ion-enriched and -depleted output channels are divided in a ratio of 3:1 in ICP Quarter (ICP_Q_), while two output channels are evenly divided in ICP Half (ICP_H_), keeping the intermembrane distance (*w*).

Under high-voltage application, electroconvection, which is a main mechanism of ion transfer in the overlimiting regime, occurs due to membrane heterogeneity, and one can approximately suppose ion transfer via the visualization of electroconvective flow [17,29,30,31]. R. Kwak et al. [32] and J. Choi et al. [25] established the scaling law of electroconvection depending on the operating parameters and fluidic channel geometry as shown below:

(1) $$\frac{D_{EC}}{w}∼C{\left(\frac{V^{2}}{U_{HP}}\right)}^{1/3},C={\left[\frac{\varepsilon }{64\mu w^{3}}\left(\frac{2wh}{w+h}\right)\right]}^{1/3}$$

where *D_EC_* is the thickness of electroconvection, while *w* and *h* are the width and height of the flow channel (i.e., intermembrane distance), respectively. *C* is a scaling constant, while *V* and *U_HP_* are the applied voltage and flow velocity, respectively. *ε* and *μ* are the electric permittivity and dynamic viscosity of the fluid, respectively. It is reasonable to assume the ion depletion zone and salt removal observing electroconvective flow since the size of electroconvection (*D_EC_*) is typically similar to the ion depletion zone. Hence, it is rational to match the ion depletion zone and the recovery of desalted flow in a low-concentration desalination.

In electromembrane desalination, the salt removal ratio (SRR), current efficiency (CE), and energy per ion removal (EPIR) can be calculated to evaluate performance and efficiency. The SRR, CE, and EPIR are defined as follows:

(2) $$Salt\;Removal\;Ratio\;\;(SRR)=\frac{C_{0}-C_{desalted}}{C_{0}}$$

(3) $$Current\;Efficiency\;\;(CE)=\frac{zFQ_{desalted}(C_{0}-C_{desalted})}{NI}$$

(4) $$Energy\;Per\;Ion\;Removal\;(EPIR)=\frac{IV/Q_{desalted}}{zk_{B}T(C_{0}-C_{desalted})}$$

where *C_0_* and *C_desalted_* are the molar salt concentrations in the feed and the desalted solution, respectively. *z*, *F*, and *N* are the ion valence, Faraday constant (= 9.65 × 10^4^ C∙mol^−1^), and number of membrane pairs (i.e., cell number), respectively; *Q_desalted_* is the volumetric flow rate in the desalination channel; and *I* is the measured current. *k_b_* and *T* are the Boltzmann constant (1.380 × 10^−23^ J∙K^−1^) and the temperature, respectively. Regarding the number of membrane pairs, it means the minimum unit that performs basic functions for desalination. For example, feed water has to be split into desalted and concentrated streams in the unit of the membrane pairs in the electromembrane process. In the case of electrodialysis, three membranes (i.e., two fluidic channels, 2Q) are necessary for the minimum unit, which is equivalent to *N* = 1. In the case of ICP desalination, however, only two membranes (i.e., one fluidic channel, Q) are necessary for the minimum unit, which is equivalent to *N* = 0.5. In other words, it can be considered that *N* = 0.5 in ICP desalination is calibrating halved the desalted flow rate (*Q_desalted_*/2) compared with electrodialysis (*Q_desalted_*). Nevertheless, the *N* value of the recovery-reduced ICP strategy must be the same as common ICP desalination since it only depends on the membrane arrangement (i.e., electrodialysis or ICP), not on the recovery. CE indicates how efficiently current is used to remove ions from desalted streams, and EPIR is the energy consumed when removing one ion.

### 2.2. Materials and Device

**Figure 2:**
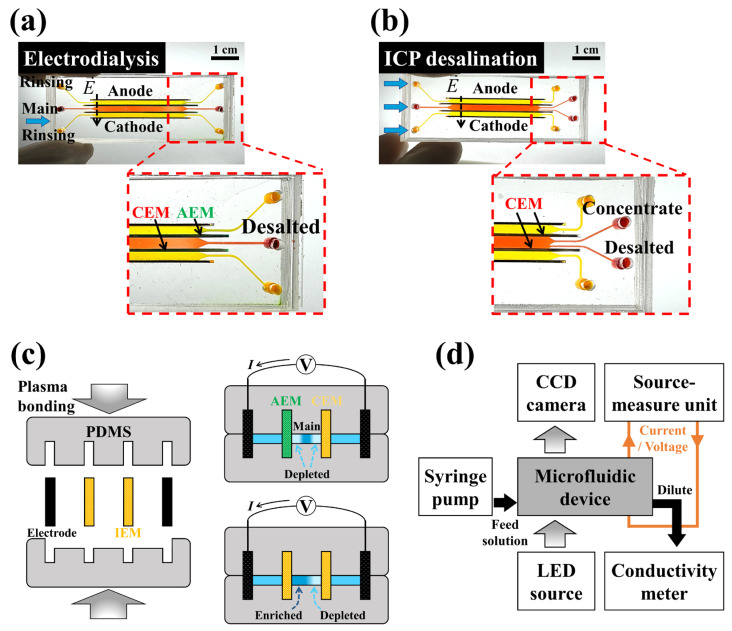
Photographs of PDMS-based microfluidic (**a**) electrodialysis and (**b**) ICP devices. Each device has two ion exchange membranes (AEM and CEM for electrodialysis and two CEMs for ICP desalination) and two carbon-based electrodes. (**c**) Schematics of the assembly of the microfluidic device. It was fabricated by mounting two carbon electrodes and two membranes, and subsequent plasma bonding to the upper and bottom PDMS blocks. (**d**) Experimental setup of the microfluidic electromembrane system for fluorescent visualization and the measurement of desalination.

To visualize electroconvective flow and the ion depletion zone near the membrane surface, we used a microscale electromembrane platform as described in previous studies [21,25,32,33]. Electrodialysis consists of two rinsing channels and one main channel with a depth of 200 μm, and those channels are separated by the AEM (Ralex AMHPP; MEGA, Prague, Czech Republic), CEM (Ralex CMHPP; MEGA, Prague, Czech Republic), and two electrodes (Spectracarb 2050A-1535; Fuel Cell Store, Bryan, TX, USA) (See Figure 2a). In the same manner, an ICP system has two rinsing channels and one main channel, but they are separated by two CEMs (See Figure 2b). The microfluidic device was fabricated by bonding upper and bottom polydimethylsiloxane (PDMS) blocks, which have narrow and deep slots for membranes and electrodes, as shown in Figure 2c. The PDMS mold was produced using a high-precision 3D printer (SLA ProJet 7000 HD; 3D Systems, Rock Hill, SC, USA). The width (*w*) and length of the microfluidic channels are 2 mm and 30 mm, respectively, while the width of the desalted outlets of ICP_H_ and ICP_Q_ are 1 mm and 0.5 mm, respectively. More operating and geometric parameters are summarized in Table 1. Low-concentration sodium chloride solutions (10 mM, 1 mM, and 0.1 mM) were injected in the main channel, while equivalent low-concentration sodium sulfate solutions (5 mM, 0.5 mM, and 0.05 mM) were used in the rinsing channels. A small amount (20 μM) of fluorescent dye (Alexa Fluor 488; Invitrogen, Carlsbad, CA, USA) was added to the solution to visualize the electroconvective flow and ion depletion zone, and a pH indicator (Hydrion one drop indicator solution; MICROESSENTIAL INC., New York, NY, USA) was used to observe pH changes.

### 2.3. Measurements

As shown in Figure 2d, the fabricated microfluidic device was connected to a syringe pump (Fusion 200-X; Chemyx, Stafford, TX, USA) to apply pressure-driven flow, keeping the shear flow velocity at 1 mm/s in all experiments. The current–voltage response was measured using a source measure unit (2460 SourceMeter; Keithley, Cleveland, OH, USA), and the concentration of desalted water was measured using a flow-through conductivity meter (16–900 Flow-thru Conductivity Electrodes; Microelectrodes, Bedford, NH, USA). The fluidic channel was monitored by a fluorescence microscope (CKX 53; Olympus, Tokyo, Japan). Currents were measured by sweeping voltage from 0 to 10 V at 0.2 V intervals (30 s) for the I-V curves, and constant voltages (2 V, 5 V, 10 V, and 15 V) were applied for 20 min in desalination experiments. The captured fluorescent images were analyzed to calculate the ion depletion zone using Image J (National Institutes of Health, Loci, WI, USA) software.

## 3. Results and Discussion

### 3.1. Current–Voltage Response

**Figure 3:**
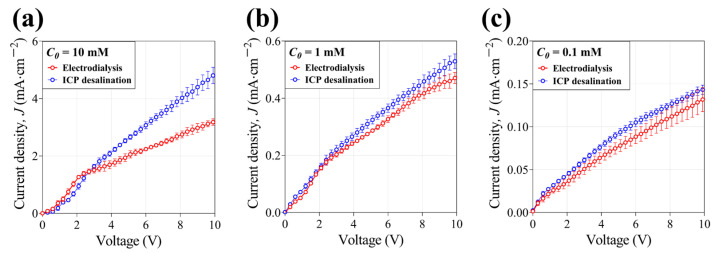
Current density–voltage (*I*–*V*) curves with (**a**) 10 mM, (**b**) 1 mM, and (**c**) 0.1 mM NaCl feed in electrodialysis and ICP desalination.

In an electromembrane system, the current (*I*)–voltage (*V*) curve consists of an Ohmic regime, where the current response increases linearly as the applied voltage increases; a limiting regime, where the current does not increase due to concentration polarization across the membrane; and the overlimiting regime, where the current increases again as the applied voltage increases [34,35]. There have been various debates on the reason for the emergence of the overlimiting regime over several decades, but the formation and development of electroconvective vortices on the membrane surface have been accepted as an established theory. In detail, the membrane heterogeneity makes small unstable vortices that enhance ion transfer again as the applied voltage increases [36,37]. As shown in Figure 3a (10 mM NaCl), in the Ohmic regime (<2 V), the ICP system exhibits fewer currents than electrodialysis, which is mainly due to the diffusivity difference of cation (*D_Na+_* = 1.33 (10^−9^ × m^2^ × s^−1^)) and anion (*D_Cl−_* = 2.03 (10^−9^ × m^2^ × s^−1^)) and the relatively higher electrical resistance of the CEM [21,35]. However, as voltage increases in the overlimiting regime, the ICP system shows higher currents than electrodialysis. This is because electroconvective vortices, which are the main mechanism of ion transfer in the overlimiting regime, are formed more strongly on the CEM. Furthermore, it is also known that the electroconvection is suppressed on the AEM due to the catalytic activity of the AEM surface [26,27,28]. As feed concentration decreases (see Figure 3b,c), it is observed that the ICP system shows higher electrical conductance in all regimes, although the difference is not large. Therefore, in the case of low-concentration feed, ICP desalination provides more current and ion transfer, which is advantageous to the desalination operation.

### 3.2. Real-Time Monitoring of Desalination with Flow Visualization

**Figure 4:**
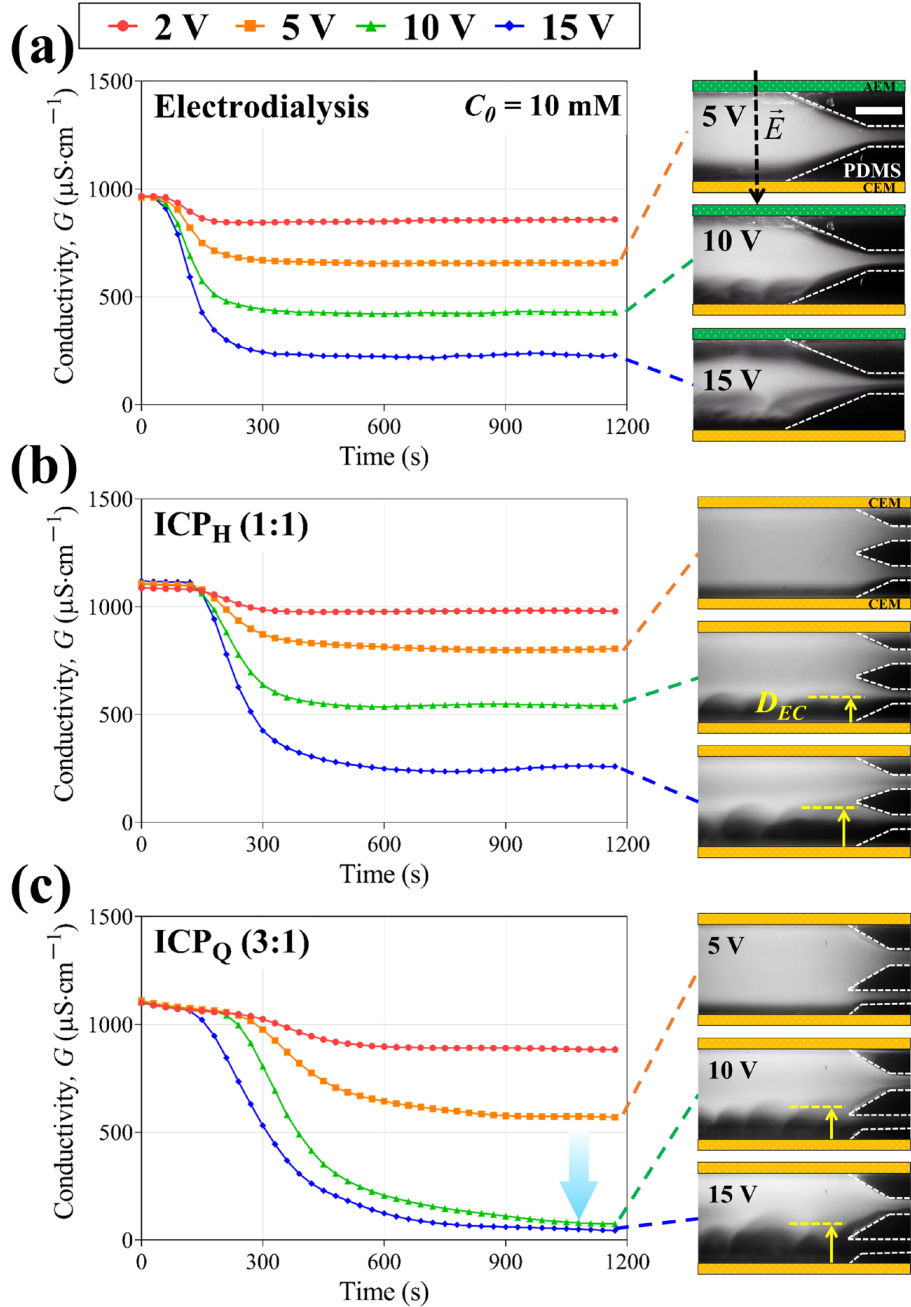
Conductivity changes in the desalted flow from 2 V to 15 V in (**a**) electrodialysis, (**b**) ICP_H_, and (**c**) ICP_Q_. Corresponding fluorescent images are shown on the right; 10 mM NaCl feed was used in all experiments; and the scale bar indicates 1 mm.

Three types of electromembrane desalination systems (electrodialysis, ICP_H_, and ICP_Q_) were operated to desalinate low-concentration feed water under constant voltage (2 V, 5 V, 10 V, and 15 V). We measured conductivity changes and monitored electroconvective flow simultaneously, as shown in Figure 4. In the fluorescent images, it is observed that the ICP system has one ion depletion zone (dark region) on the bottom and one ion enrichment zone on the top, while electrodialysis has two ion depletion zones on both the CEM and AEM. When the electroconvection is identified at high voltage (>5 V), the thickness of the electroconvective boundaries (*D_EC_*) can be estimated via the brightness of the fluorescent dye.

Indeed, the thickness of electroconvection (*D_EC_*) could be different from that of the ion depletion zone due to different ionic mobilities between sodium chloride and fluorescent dyes, but we simply assume that those boundaries are not much different so that we can regard the thickness of electroconvection (*D_EC_*) as an ion-depleted domain [32,33].

**Figure 5:**
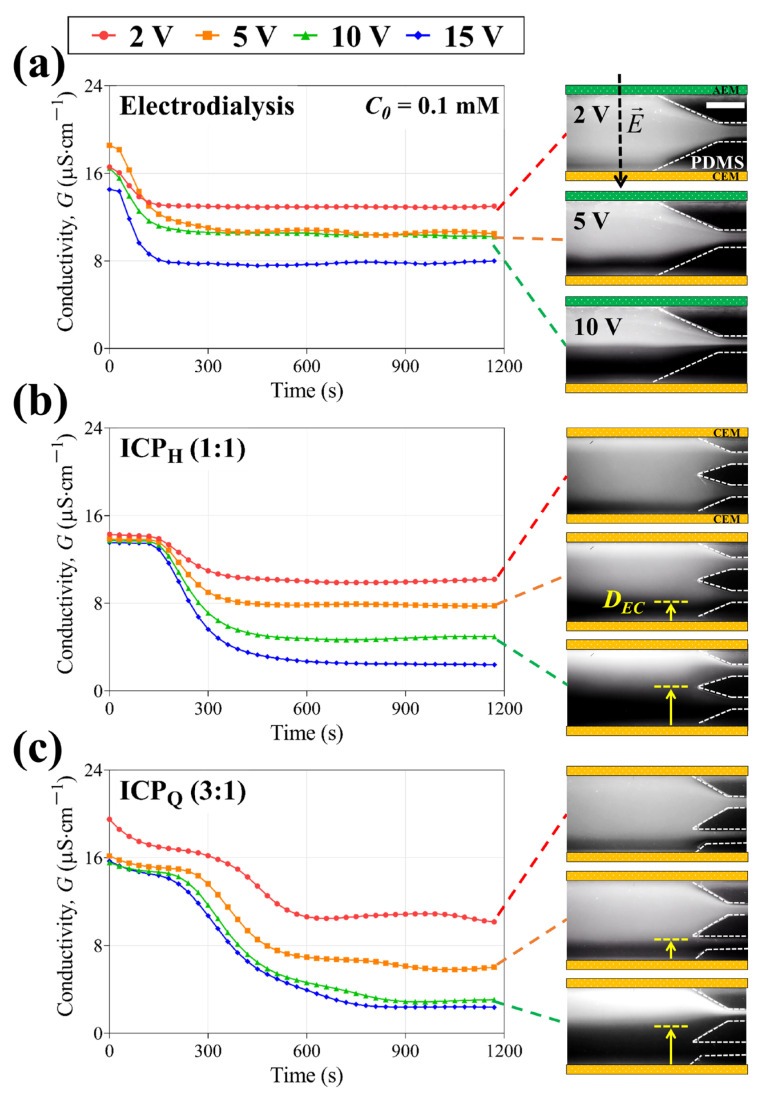
Conductivity changes in the desalted flow from 2 V to 15 V in (**a**) electrodialysis, (**b**) ICP_H_, and (**c**) ICP_Q_. Corresponding fluorescent images are shown on the right; 0.1 mM NaCl feed was used in all experiments; and the scale bar indicates 1 mm.

Figure 4 shows real-time conductivity changes in the 10 mM NaCl feed, and conductivity drops were observed linearly proportional to the applied voltage in both electrodialysis and ICP_H_. Contrastively, a rapid conductivity drop was measured between 5 V and 10 V, where the thickness of the electroconvection becomes similar to the outlet width. This shows that a much higher salt removal rate can be achieved by simple outlet modification in ICP desalination. For the 0.1 mM NaCl feed (See Figure 5), due to the very low concentration, the behavior of the fluorescent dye is no longer an indicator of the electroconvective flow but acts as a charge carrier. In single-step electrodialysis, it is difficult to achieve a higher SRR (>50%) since it is typically operated in the Ohmic regime. Moreover, even though the applied voltage increases (i.e., the overlimiting regime), electroconvection on the AEM could weaken, which arises from proton generation on the AEM surface. In ICP desalination, although ions looked concentrated by electric field-induced migration, a high salt removal rate was observed when the thickness of the electroconvection was similar to the outlet width, as confirmed by the 10 mM NaCl feed (Figure 4b,c) [32,33,38]. After all, ICP desalination exhibits a high SRR of more than 90%, while electrodialysis shows an SRR of less than 50% in low-concentration (0.1 mM NaCl) desalination. Based on the experiments, it can be found that the ICP strategy is more facile to achieve complete desalination in low-concentration feed. 

### 3.3. Desalination Metrics

**Figure 6:**
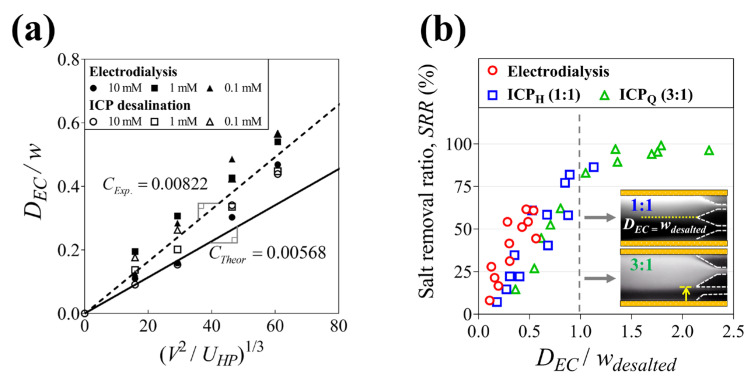
(**a**) Dimensionless thickness of the electroconvection zone (*D_EC_*/*w*) plotted against the scaling factor ((*V^2^*/*U_HP_*)^1/3^) at various applied voltages and feed concentrations in electrodialysis and ICP desalination. The black line and the dotted line indicate the scaling law by the theory and experiments, respectively. (**b**) The salt removal ratio depending on the electroconvection zone (*D_EC_*/*w_desalted_*).

We measured the thickness of electroconvection (*D_EC_*) via fluorescent visualization in the microfluidic electromembrane desalination and summarized the operating parameters (*V* and *U_HP_*) to verify the scaling law described in Section 2.1. As shown in Figure 6a, it is observed that the electroconvection zone (*D_EC_*/*w*) linearly expands as the scaling factor ((*V^2^*/*U_HP_*)^1/3^) increases. Nevertheless, the slope (0.0082, dotted line) of the *D_EC_* measurement is larger than the slope (0.00568, line) of the *D_EC_* calculation. This is because the feed concentrations used in the experiments are relatively low-enhancing electroconvection. Based on the analysis, it is possible to control the ion depletion zone via the operating parameters to match a target SRR in ICP desalination. As described, ICP desalination has the unique characteristics of utilizing thin ion-depleted flow formed on the membrane surface. Consequently, the thickness of electroconvection (*D_EC_*) and the width of the desalted outlet (*w_desalted_*) have a very strong correlation with the SRR. Hence, we already observed that the SRR increases the most when *w_desalted_* becomes close to *D_EC_*, as explained in Section 3.2. In addition, we indicated the electroconvection zone (*D_EC_*/*w_desalted_*) and the SRR in all electromembrane desalination types to readily comprehend their relationship (See Figure 6b). Since electrodialysis has only one outlet (i.e., *w*~*w_desalted_*), it could be assumed that the SRR increases as voltage increases. In ICP desalination, however, the SRR also increases until *D_EC_* is shorter than *w_desalted_*, but it is rapidly saturated when *D_EC_* becomes larger than *w_desalted_*. Accordingly, for up to 80% of the SRR in low-concentration desalination, one can incorporate a highly efficient desalination system based on the scaling law in recovery-reduced ICP desalination (e.g., ICP_Q_). Furthermore, it should be noted that the maximum SRR of electrodialysis and ICP_Q_ are 62% and 99%, respectively. Given that we pursue the SRR as much as possible in the applications of low-concentration desalination, recovery-reduced ICP desalination could be more useful than electrodialysis. 

**Figure 7:**
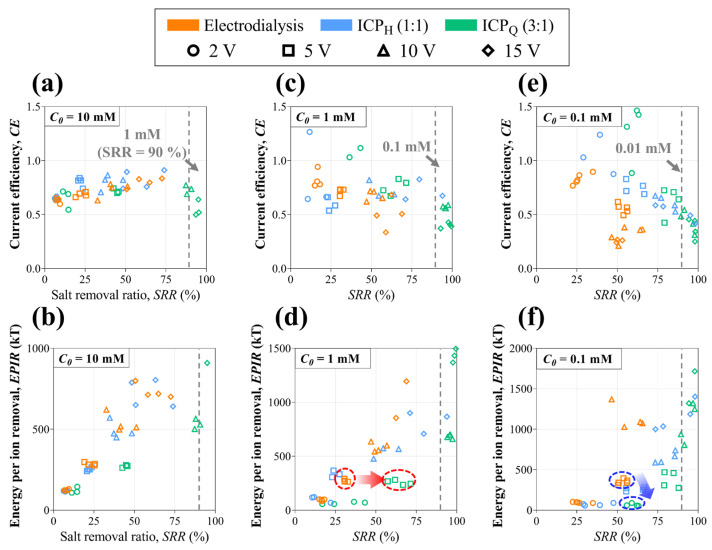
Current efficiency (CE) and energy per ion removal (EPIR) for (**a**,**b**) 10 mM, (**c**,**d**) 1 mM, and (**e**,**f**) 0.1 mM NaCl feed. The gray dotted line indicates SRR 90%.

To examine desalination efficiency, CE and EPIR depending on the SRR are calculated based on the experimental data of all the types in Figure 7. In the case of NaCl 10 mM, CE (See Figure 7a) is at a similar level for all types, but ICP_Q_ shows lower CE at high voltage (10 V and 15 V). It is mainly because ion ion-depleted stream includes an enriched outlet where it achieves a much higher SRR (~90%) than the other types. Looking at the EPIR plot (See Figure 7b), ICP_Q_ exhibits lower EPIR until 90% of the SRR but shows a significant increase in EPIR at a high applied voltage (15 V) and SRR (>90%). As the feed concentration decreases (NaCl 1 mM and 0.1 mM), ICP types achieve a high SRR at a relatively low applied voltage, while electrodialysis cannot reach those high SRRs even though a high voltage is applied (See Figure 7c–f). Hence, ICP desalination can be confirmed in applications that require a high SRR in low-concentration feed. In particular, ICP_Q_ shows ab SRR that is more than twice as high compared with that of electrodialysis at the same applied voltage (see the red circles in Figure 7d) and EPIR is less than half as low compared with electrodialysis at the same SRR (see the blue circles in Figure 7f). Even so, it has to be mentioned that EPIR can dramatically increase when desalted streams pass over the width of the desalted outlet (i.e., SRR > 99%). Therefore, utilizing single-step recovery-reduced ICP desalination, one can realize much a higher SRR with less EPIR by optimizing outlet recovery and operating parameters for various feed concentrations.

### 3.4. Observation of pH Changes

**Figure 8:**
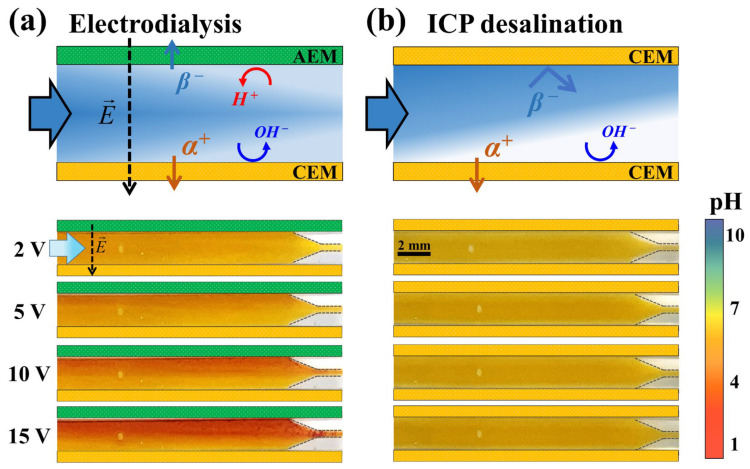
Observation of pH changes in (**a**) electrodialysis and (**b**) ICP desalination applying voltages from 2 V to 15 V. 0.1 mM NaCl solution used as feed, with a pH value ranging from 6 to 7.

One of the significant differences between electrodialysis and the ICP strategy is the presence or absence of an AEM. It is known that water splitting occurs more actively on the AEM surface since the catalytic activity of the fixed charge group in an AEM is greater than that of the fixed charge group in a CEM [26,27,28]. The generated H^+^ and OH^−^ interfere with the development of electroconvective vortices, thus hindering ion transfer. To confirm the phenomena more clearly, we visualized a pH shift in the flow in the electrodialysis and ICP devices using a pH indicator. As shown in Figure 8, the pH shift in electrodialysis occurs around 6–7 through the whole channel, whereas in ICP, it occurs around 0–1 near the ion depletion zone at the same applied voltage. In other words, electrodialysis has a significant loss due to water splitting rather than ion transfer in low-concentration desalination. This agrees with the experimental results that electrodialysis has a lower SRR and CE and a higher EPIR than ICP desalination.

## 4. Conclusions

Low-concentration desalination is an essential technology for producing ultrapure water, where a higher SRR is a more important criterion than energy efficiency. In this study, we compared electromembrane desalination technologies such as electrodialysis and the ICP strategy for low-concentration desalination. In addition, we developed a recovery-reduced ICP, which has a narrow width of the desalted outlet for a higher SRR. For three types of electromembrane desalination (electrodialysis, ICP_H_, ICP_Q_), we found important results based on the fluorescent visualization and desalting performances as follows. First, under single-step desalination and similar operating conditions, the SRR was mostly around 50% for electrodialysis but was high enough to exceed 90% for recovery-reduced ICP (ICP_Q_). Although ICP_Q_ has losses in recovery, it has the obvious advantage of being able to obtain water of much higher purity. Second, recovery-reduced ICP desalination showed a high SRR close to 100% at the moment the width of the desalted outlet became similar to the thickness of the ion depletion zone. Considering that forming an ion depletion zone up to half the channel width in the electromembrane system generally requires a very large amount of power consumption, an ICP strategy that can adjust the width of the desalted outlet for a high SRR is recommended. Lastly, when overlimiting current is applied, many pH changes near the AEM are observed in electrodialysis, which inhibits electroconvection and makes it difficult to achieve a high SRR. Therefore, for complete desalination to produce ultrapure water, ICP desalination utilizing only a CEM could be more valid than electrodialysis. Here, we examined the low-concentration desalination of electrodialysis and ICP desalination. Although there are specialized technologies for producing ultrapure water such as CEDI, recovery-reduced ICP desalination can be a potentially valuable and preferable technology that can readily achieve a high SRR without using additive ion exchange resins.

## Figures and Tables

**Table 1:** Parameters in electrodialysis, ICP_H_, and ICP_Q_. *Q_main_* and *Q_desalted_* indicate the feed and desalted flow rates, respectively. *A_desalted_* is a cross-sectional area of the microfluidic desalted channel.

Type	Flow Velocity, *v* [mm/s]	*Q_main_* [mL/min]	*A_desalted_* [mm^2^]	*Q_desalted_* [mL/min]
Electrodialysis	1	24	0.4	24
ICP_H_ (1:1)			0.2	12
ICP_Q_ (3:1)			0.1	6

## Data Availability

The original contributions presented in the study are included in the article, further inquiries can be directed to the corresponding authors.
